# Identification of Astrovirus in the virome of the upper and lower respiratory tracts of calves with acute signs of bronchopneumonia

**DOI:** 10.1128/spectrum.03026-23

**Published:** 2023-11-20

**Authors:** Maria Gaudino, Elias Salem, Mariette F. Ducatez, Gilles Meyer

**Affiliations:** 1 IHAP, Université de Toulouse, INRAE, ENVT, Toulouse, France; Changchun Veterinary Research Institute, Changchun, China

**Keywords:** Astrovirus, bovine respiratory disease, NGS, metagenomics, phylogeny

## Abstract

Bovine Astroviruses (BAstV) are small single-stranded, positive-sense RNA viruses that have been historically associated with the infection of the enteric tract in cattle, causing outbreaks of diarrhea, especially in young immunologically naive calves. Long neglected, BAstV have recently regained interest as they have been associated with several nonsuppurative encephalitis cases in cattle as well as in other species. Yet, the difficulty in isolating the virus in conventional cell cultures prevents a full understanding of BAstV tropism and pathogenesis in this species. Here, we describe the metagenomic identification of BAstV in the respiratory tract of calves affected by respiratory distress in French farms. BastV were prevalently detected in the lower respiratory tract of sick calves; however, molecular testing of further cohorts of respiratory samples highlighted their presence also in nasal swabs. Phylogenetic analyses revealed two divergent clades for neurotropic and enteric BAstV, suggesting that their tropism is likely to be strain-specific. BAstV detected in the respiratory tract of calves with respiratory disease were genetically related to enteric strains already described in cattle in Asia, displaying between 85% and 95% nucleotide identity. In addition, the sequences from France belonged to the clade of BAstV identified in yaks, roe deer, and camelids, underlining the interspecies transmission potential of the virus. More studies are warranted to understand if BAstV have a potential role in the onset of bovine respiratory disease.

**IMPORTANCE:**

Astroviruses (AstV) are known suspects of enteric disease in humans and livestock. Recently, AstV have been linked to encephalitis in immunocompromised patients and other animals, such as cattle, minks, and swine. In our study, we also identified AstV in the respiratory samples of calves with signs of bronchopneumonia, suggesting that their tropism could be even broader. We obtained one bovine AstV (BAstV) complete genome sequence by next-generation sequencing and showed that respiratory and enteric AstV from different species formed a divergent genetic cluster with AstV isolated from encephalitis cases, indicating that tropism might be strain-specific. These data provide further insight into understanding the biology of these understudied pathogens and suggest BAstV as a potential new candidate for bovine respiratory disease.

## INTRODUCTION

Astroviruses (AstV) are small non-enveloped viruses belonging to the *Astroviridae* family with a non-segmented, positive sense, single-stranded RNA genome and an icosahedral capsid (1). They are approximately 28–35 nm in diameter, their genome is between 6.8 and 7.9 kb, and the uncapped RNA at 3′ untranslated region (UTR) is linked to a poly(A) tail, whereas the 5′ UTR is linked to a viral genome-linked protein (VPg) (2). The AstV genome is arranged into three overlapping open reading frames (ORFs). ORF1a encodes non-structural proteins that are cleaved by viral proteases, while ORF1b encodes the RNA-dependent RNA polymerase. ORF2 encodes the capsid precursor, which undergoes multiple cleavages by host proteases (3). In particular, VP90 (the full-length precursor) is cleaved by cellular caspases to generate VP70; however, the virions remain non-infectious until further cleaved to generate mature viral particles containing VP25, VP27, and VP34 polypeptides on the surface (4). In addition, an alternative frame ORF (named ORF-X) within the ORF2 has recently been described and its expression is linked to viroporin-like activity (5).

Based on the current ICTV taxonomy, the AstV family is divided into two genera: *Avastrovirus*, causing hepatitis, nephritis, and diarrhea in birds, and *Mamastrovirus,* infecting mammals (6). Once thought to be species-specific, evidence suggests that AstV can spread between different hosts, primarily relying on fecal-oral transmission (7). Their cross-transmission is facilitated by their high genetic variability and their high frequency of recombination events (8). Although the field of AstV research is small and limits the understanding of their biology and pathogenesis, the recent advance in technologies has allowed its expansion (9). *Mamastrovirus* has a broad susceptible host range, including humans, domestic (cattle, swine, sheep, rabbits, cats, dogs, and minks), and wild animals (bats, mice, rats, foxes, marmots, dromedaries, deer, dolphins, and sea lions) (10). AstV pathogenesis has been primarily linked to enteric disease in most of the species; however, upon AstV infection several cases of encephalitis have also been described. In addition, *Avastrovirus* can cause nephritis and hepatitis in birds (11, 12).

Bovine Astroviruses (BAstV) were discovered in 1978 following the isolation of small round viral particles from the feces of British diarrheic calves (13). Long neglected, BAstV have regained interest in the last decade (14), and they have been associated with enteritis (15 – 18) and encephalitis (19 – 21). So far, BAstV pathogenesis in cattle has been evaluated based on viral detection in animals displaying clinical signs. The difficulty of isolating the virus *in vitro* has prevented researchers from better understanding BAstV pathogenesis, as the Koch’s postulate proof-of-concept challenge of the causative agent into healthy experimental animals to reproduce the disease has yet to be carried out.

Unlike human AstV, BastV remain understudied today. In addition to the lack of isolation in cell culture, the coinfection with other pathogens also limits the understanding of their impact on cattle health. While in the literature BAstV detection has been described as limited to the digestive and nervous systems, two case-control studies conducted in the USA and Canada reported BAstV detection in the nasal swabs (NS) of calves with respiratory signs. However, this finding was not significantly associated with respiratory disease compared to healthy controls (22, 23).

Here, we describe BAstV detection in calves’ upper and lower respiratory tracts during respiratory disease outbreaks in different farms in South-West France. Different cohorts of bronchoalveolar lavages (BAL) and NS that were submitted to Illumina next-generation sequencing (NGS) sequencing revealed the presence of BAstV by metagenomics analysis. In addition, we obtained partial and complete genome sequences and carried out phylogenetic analyses to better characterize the circulating strains of BAstV detected in the respiratory tract.

## MATERIALS AND METHODS

### Clinical samples

Clinical samples included BAL and NS and were collected during veterinary check-ups in calves’ farms with respiratory disease outbreaks. The farms were located in two French regions: Occitanie (South-West of France), 150 km radius from the city of Toulouse, and Bretagne (North-West of France), 70 km radius from Carhaix-Plouguer. The farm locations are depicted in Fig. S1. In total, calves (*n* = 96) from 23 farms (three to five samples per farm) were sampled in Occitanie during the winter seasons from 2013 to 2016. In 2018, nasal swabs were collected from 72 calves on three farms. In Bretagne, samples were collected from 16 calves on one farm in January 2020, and 67 calves were sampled on 10 different farms in the winter season 2020–2021. The calves were maximum 4 months of age and displayed hyperthermia and acute signs of respiratory disease. The animals were either non-vaccinated or did not receive vaccination for at least 3 weeks prior to sampling. Details about the sample collection protocol have been previously described (24).

### Next-generation sequencing

NGS sequencing was carried out on the cohort of samples collected from 23 farms in the Occitanie region in the winter seasons from 2013 to 2016. For NGS sequencing, 23 pools of BALs and 23 pools of NS (one for each farm) were created and for each pool three to five animals were included. BAL pools were created by adding 1 mL of sample/animal, whereas NS pools were created by adding 100 µL of sample/animal. Subsequently, 300–500 µL of NS pools and 3–5 mL of BAL pools were filtered at 0.45 µm, and the viral particles were concentrated at 100,000 *g* for 2 hours at +4°C. The pellets were resuspended in a final volume of 300 µL and then treated with a cocktail of nucleases to eliminate naked DNA and RNA using Exonuclease I (20 U, Thermo Fisher), Benzonase (25 U, Merck), Turbo DNAse (2 U, Thermo Fisher Scientific), and RNase I (10 U, Thermo Fisher) and incubated for 1 hour at 37°C. The remaining nucleic acids were extracted using the Qiamp minElute virus spin kit (Qiagen) and tagged random octamers (Tag-D CGTAGATAAGCGGTCGGCTC and Tag-E CATCACATAGGCGTCCGCTG) were used for RT-PCR. Klenow fragment polymerase (New England Biolabs) was then used to perform a single round of double-strand DNA synthesis. Libraries were created using the TrueSeq nano kit (Illumina) and sequenced on a HiseqTM 2500 platform (Illumina), generating 2 × 250 reads. NGS libraries were screened for BAstV presence using the virus-integrated pipeline (VIP) implemented on Galaxy workbench (25, 26). In our work, we set a cut-off of at least 10 reads for each identified virus to consider the result reliable. Contigs were assembled using Burrows-Wheeler Alignment tool (v.0.7.12-r1039) implemented on Galaxy workbench (25) using different reference genomes for assembly: BoAstV/JPN/Ishikawa24-6/2013, an enteric BAstV (GenBank accession number: LC047787.1) and BastV/CH13 and BastV/CH15, two neurotropic BastV (GenBank accession numbers: KM035759.1 and KT956903.2). The assembled reads were visualized using Geneious Prime v2023.0.4. ORFs were predicted using NCBI ORF finder (https://www.ncbi.nlm.nih.gov/orffinder).

### Molecular screening of enteric BAstV and other BRD pathogens by quantitative reverse transcription PCR

The samples were screened after collection by quantitative reverse transcription PCR (RT-qPCR) using the VetMAX Screening pack-Ruminant Respiratory Pathogens real-time PCR kit (Thermo Fisher), following the manufacturer’s protocol. For BAstV screening of clinical samples and relative quantification of viral loads, we developed an RT-qPCR detection assay based on the sequences of enteric BAstV. Briefly, the viral RNA was isolated from 150 µL/sample using the QIAamp viral RNA minikit (Qiagen), following the manufacturer’s instructions. Publicly available BAstV enteric sequences were downloaded from GenBank and aligned using ClustalW on BioEdit v7.2.5 (27). Primers for molecular screening by RT-qPCR were designed on a conserved region of the RNA-dependent RNA polymerase (Rdrp), using Primer3web v4.1.0 (28, 29): forward: 5′-TGTGGGTCAAACCTGAGAAAG-3′ and reverse: 3′-GATTGGATGGTACTGGCTGATAG-5′. The RT-qPCR was performed using the iTaq Universal SYBR Green One-step kit (Bio-Rad) using the amplification protocol of the manufacturer. The reaction was run on a LightCycler 96 real-time PCR system (Roche), and the results were analyzed using the LightCycler 96 Software version 1.1.01320 (Roche). The specificity of the amplicons was verified with melting curve analysis using samples previously sequenced by NGS as positive controls.

### Detection of BAstV in clinical samples by conventional PCR and Sanger sequencing

In addition to the RT-qPCR assay, a broadly reactive primer pair targeting a small region (427 bp) of the Rdrp within the ORF1b (MA4 and MA2) (30) was used for the screening of clinical samples. Five microliters of RNA/sample was reverse transcribed using the MA2 primer and the RevertAid RT Reverse Transcription Kit (Thermo Scientific). The amplification was then carried out in 2 µL of cDNA using the primers MA4 and MA2 (at a final concentration of 2.5 µM for each primer) and the Phusion Hot Start II High-Fidelity DNA Polymerase (Thermo Scientific), using the manufacturer’s protocol. The PCR products were purified using the NucleoSpin Gel and PCR Clean-up kit (Macherey-Nagel) and sequenced with Sanger technology (Eurofins, GATC).

### Virus isolation

Attempts of viral isolation were performed for samples with the lowest Cq values (between 18 and 21) on multiple cell lines in 24-well plates in Opti-MEM reduced serum medium (Thermo Fisher Scientific) in the presence of tosylsulfonyl phenylalanyl chloromethyl ketone trypsin (1  µg/mL, Thermo Fisher Scientific), amphotericin B (2.5  µg/mL, Sigma-Aldrich), and 1% penicillin-streptomycin (10,000 U/10  mg/mL, Pan Biotech). Virus isolation was attempted on epithelial cell lines of bovine origin (MDBK, EBL2, BT, and KOP-R) and non-bovine origin (hRT-18g, ST, Vero E6, and MDCK). One hundred fifty microliters/well of sample was inoculated on 70%–80% confluent monolayers. The inoculum was incubated for at least 2–3 hours at 37°C with 5% CO2 and then removed, following two washing steps with phosphate-buffered saline. The cells were observed daily under a light microscope to assess the presence of cytopathic effects (CPEs). The virus replication was investigated by RT-qPCR on the infected supernatants at 3-, 5-, and 7-days post-infection (p.i.). In parallel, viral isolation was also investigated after two blind passages with 5 days of incubation each.

### Recombination detection and phylogenetic reconstruction

Publicly available BAstV whole genome sequences were downloaded from NCBI. BAstV sequences were aligned using Clustal Omega implemented on the European Molecular Biology Laboratory-EBI search and sequence analysis tools (31, 32). Recombination events were searched using the Genetic Algorithm of Recombination Detection method (GARD) (33) in the Datamonkey server of HyPhy v2 (34, 35). Maximum-Likelihood phylogenetic trees were generated on the ORF1b (3,202–3,716) and a partition of the ORF2 hypervariable region (4,017–4,935) using the general time reversible model in MEGA-X v10.1.7, and the tree robustness was assessed by 1,000 bootstrap replicates (36).

## RESULTS

### BAstV detection in the lower respiratory tract of French calves with bovine respiratory disease by Illumina metagenomic sequencing

We carried out Illumina sequencing of BAL and NS collected from 93 veal calves displaying respiratory disease in the winter seasons from 2013 to 2016. The pools were sequenced in three different runs (~15 pools per run), which generated 140, 93, and 219 million reads, respectively.

By screening the metagenome results using the VIP pipeline, we detected the following known bovine respiratory disease (BRD) viruses (in decreasing order): bovine coronavirus (BCoV), bovine respiratory syncytial virus (BRSV), and bovine parainfluenza virus type 3 (BPIV-3). Less frequently, we also detected bovine rhinitis virus A and B (BRAV and BRBV), bovine parvovirus type 2, and bovine enterovirus. We detected BRD pathogens at a higher prevalence by NGS compared to the commercial kit, suggesting that NGS had a higher analytical sensitivity compared to RT-qPCR for our samples.

Interestingly, we detected BAstV in half (*n* = 11) of the tested BAL pools (*n* = 23) (47.82%); however, we detected BastV only in one NS pool (4.34% of the tested NS pools), as shown in Table 1. BastV were in coinfection with several BRD viruses. BastV and other coinfecting viruses identified by metagenomics are also described in Table 1.

**TABLE 1:** Genome coverage, number of reads, and average depth of coverage of BAstV and other coinfecting viruses identified in BAL and NS pools by metagenomic sequencing and using a commercial kit for BRD pathogen detection^*a*^ ^,*b*^

Sample	Date of sampling	Virus identified	Coverage (%)	Number of reads	Average depth of coverage	Pathogens detected by RT-qPCR (Cq)
BAL-1	December 2013	**BAstV** BRSV BPIV-3 BCoV	**88.57** 99.28 93.41 88.30	**168** 7,724 617 1,313	**3.74** 117.99 7.61 8.06	BCoV (37.58)
BAL-3	January 2013	**BAstV** BRSV BPIV-3 BCoV	**93.78** 29.79 92.77 94.43	**291** 65 841 2,258	**6.51** 0.54 9.30 14.19	
BAL-4 (BAstV/ICSA-4/France/2013)	January 2014	**BAstV** BRSV BPIV-3 BCoV BRBV	**100.00** 23.28 99.84 71.29 64.85	**200,095** 52 6,228 573 91	**7,534.37** 0.37 97.25 3.15 2.27	BRSV (22.74), BCoV (30.17)
BAL-6	January 2014	**BAstV** BRSV BPIV-3 BCoV	**98.79** 23.30 99.98 90.37	**373** 60 875,801 1,660	**11.29** 0.44 14,590.64 9.27	
BAL-7	January 2014	**BAstV** BRSV BPIV-3 BCoV	**75.25** 25.99 93.53 89.33	**213** 63 917 1,653	**4.69** 0.45 11.33 9.86	BRSV (25.8)
BAL-9	March 2014	**BAstV** BRSV BPIV-3 BCoV BRBV	**73.61** 16.34 86.70 99.96 29.57	**88** 31 381 950,109 25	**2.12** 0.22 4.86 8,020.85 0.63	BCoV (31.11)
BAL-10	March 2014	**BAstV** BRSV BPIV-3 BCoV	**66.12** 69.40 84.40 77.56	**92** 401 341 605	**2.21** 5.90 4.13 3.54	BCoV (28.12)
BAL-11	March 2014	**BAstV** BPIV-3 BCoV	**97.28** 93.73 91.95	**318** 795 1,695	**7.46** 9.32 9.64	BPIV-3 (23.56)
BAL-15	November 2014	**BAstV** BRSV BPIV-3 BCoV	**39.68** 32.84 52.92 99.96	**43** 66 131 379,033	**0.68** 0.6986 1.10 3,149.22	
BAL-16	November 2014	**BAstV** BRSV BPIV-3 BCoV	**65.57** 34.11 56.49 93.14	**71** 67 187 2,245	**1.17** 0.62 1.55 15.56	BCoV (24.19)
BAL-17	December 2014	**BAstV** BRSV BPIV-3 BCoV	**70.80** 20.95 55.59 71.22	**154** 49 128 541	**4.14** 0.40 1.17 2.80	BCoV (38.9)
NS-18	December 2014	**BAstV** BPIV-3 BCoV BRBV	**42.77** 17.14 99.28 34.56	**134** 32 5,315 33	**3.14** 0.23 32.67 0.55	

^*a*^ The samples were collected from calves with BRD in Occitanie in the winter seasons from 2013 to 2016.

^*b*^ BAL: bronchoalveolar lavage; NS: nasal swab; BAstV: Bovine Astrovirus; BRSV: Bovine Respiratory Syncytial Virus; BPIV-3: Bovine Parainfluenza Type 3; BCoV: Bovine Coronavirus.

One pool (BAL-4) contained significant viral loads (200,095 reads), and we could retrieve a complete BAstV genome that we named BAstV/ICSA-4/France/2013, and that was used for further molecular characterization. Based on the assembly on a reference genome, BAstV/ICSA-4/France/2013 displayed a total length of 6,287 nucleotides. We did not, however, confirm the 5′ and 3′ ends with a RACE PCR approach; therefore, the untranslated regions might be longer. The ORF prediction confirmed the presence of three main overlapping ORFs (Fig. S2).Fig. S3 The ORF1a displayed a 2,454-nucleotide length (818 aa), with the highly conserved ribosomal frameshift signal 5′-AAAAAAC-3′ present at position 2,402 (within the CDS of ORF1). ORF1b displayed a nucleotide length of 1,501 bp, while ORF2 was 2,340 bp long (780 aa).

The best BLAST hits of BAstV/ICSA-4/France/2013 sequence revealed 95.91% and 85.58% nucleotide identity with the Chinese sequences BoAstV37/2021/CHN and BoAstv/CHN/HLJ-1/2019, respectively, and 84.36% with the Japanese sequence BoAstV/JPN/Ishikawa24-6/2013, as described in Table 2. Reads’ assembly using the sequence “BoAstV/JPN/Ishikawa24-6/2013” as a reference allowed for the retrieval of seven BAstV partial genomes and one complete genome. No reads were assembled when using the neurotropic sequence “BAstV/CH13” as the reference genome, suggesting the absence of neurotropic BAstV in cattle respiratory samples.

**TABLE 2:** BLASTN analysis of BAstV/ICSA-4/France/2013 complete genome

Best BLAST hit (accession number)	Best BLAST hit (country)	Best BLAST hit (host)	Best BLAST hit (isolation source)	Best BLAST hit (year)	Nucleotide identity (%)
ON682272.1	China	Cattle	Feces	2021	95.91
MW373715.1	China	Cattle	Feces	2019	85.58
LC047787.1	Japan	Cattle	Feces	2013	84.36
NC_023630.1	Hong Kong	Cattle	Feces	2008–2010	86.77
OQ198051.1	China	Dog	Feces	2020–2021	83.23
KM822593.1	China	Yak	Feces	2013	82.74
MN150125.1	Slovenia	Roe deer	Feces	2014	81.91
MT758371.1	Egypt	Cattle	Feces	2015	80.62

In addition, we then screened further cohorts of respiratory specimens collected during respiratory outbreaks between 2018 and 2021 in two different French regions by RT-qPCR. BAstV were detected in all the tested cohorts with a different prevalence depending on the sample type. BAstV presence in NS ranged from 0% to 32.83%, whereas in BAL, the positivity was between 12.5% and 28.26%. Interestingly, in two cohorts, BAstV were more prevalent in the LRT than the URT, as shown in Table 3.

**TABLE 3:** Prevalence of BAstV detected by RT-qPCR in cohorts of respiratory samples (BAL and NS) collected in France between 2013 and 2021

Year of sampling (month)	Region	Type of specimen	No. of animals (no. of herds)	No. of tested samples	No. of positive samples (%)	Rdrp sequences obtained
2013–2016 (November–March)	Occitanie	BAL; NS	93 (23 herds)	BAL: 23 pools; NS: 23 pools	BAL: 10 pools/23 pools (47.82%); NS: 1 pool/23 pools (4.34%)	BAstV/ICSA-4/France/2013 (complete genome); BAstV/3812-3/France/2013; BAstV/3837-3/France/2013; BAstV/4917-11/France/2013; BAstV/4918-11/France/2013; BAstV/4919-11/France/2013
2018 (February–March)	Occitanie	NS	72 (3 herds)	NS: 29 pools	7 pools/29 pools (24.13%)	BAstV/C4/France/2018; BAstV/C5/France/2018
2020 (January)	Bretagne	BAL; NS	16 (1 herd)	16 BAL; 32 NS (1 herd)	BAL: 3/16 (12.5%); NS: 0/30 (0%)	
2020–2021 (December–January)	Bretagne	BAL; NS	67 (10 herds)	46 BAL, 67 NS	BAL: 13/46 (28.26%); NS: 22/67 (32.83%)	BAstV/4948/France/2020; BAstV/T996/France/2020; BAstV/T990/France/2021

### Respiratory BAstV scarcely replicate *in vitro*

To better characterize respiratory BAstV, we attempted virus isolation from clinical samples (*n* = 13) on conventional cell culture monolayers of bovine and non-bovine origin. Despite multiple attempts, we failed to establish an *in vitro* model that allowed virus replication. No obvious CPEs were observed on the cell monolayers in the days following the infection. By RT-qPCR, we screened the infected supernatants to assess virus replication at 3-, 5-, and 7-days p.i.; however, only a low amount of virus was observed, with Cq values ranging from 30 to 37. Upon the second passage, all the supernatants were negative or yielded very high Cq values, suggesting that conventional protocols and cell lines used in this study are inadequate for culturing BAstV.

### BAstV detected in the respiratory tract of calves with BRD are genetically close to enteric strains

**Fig 1:**
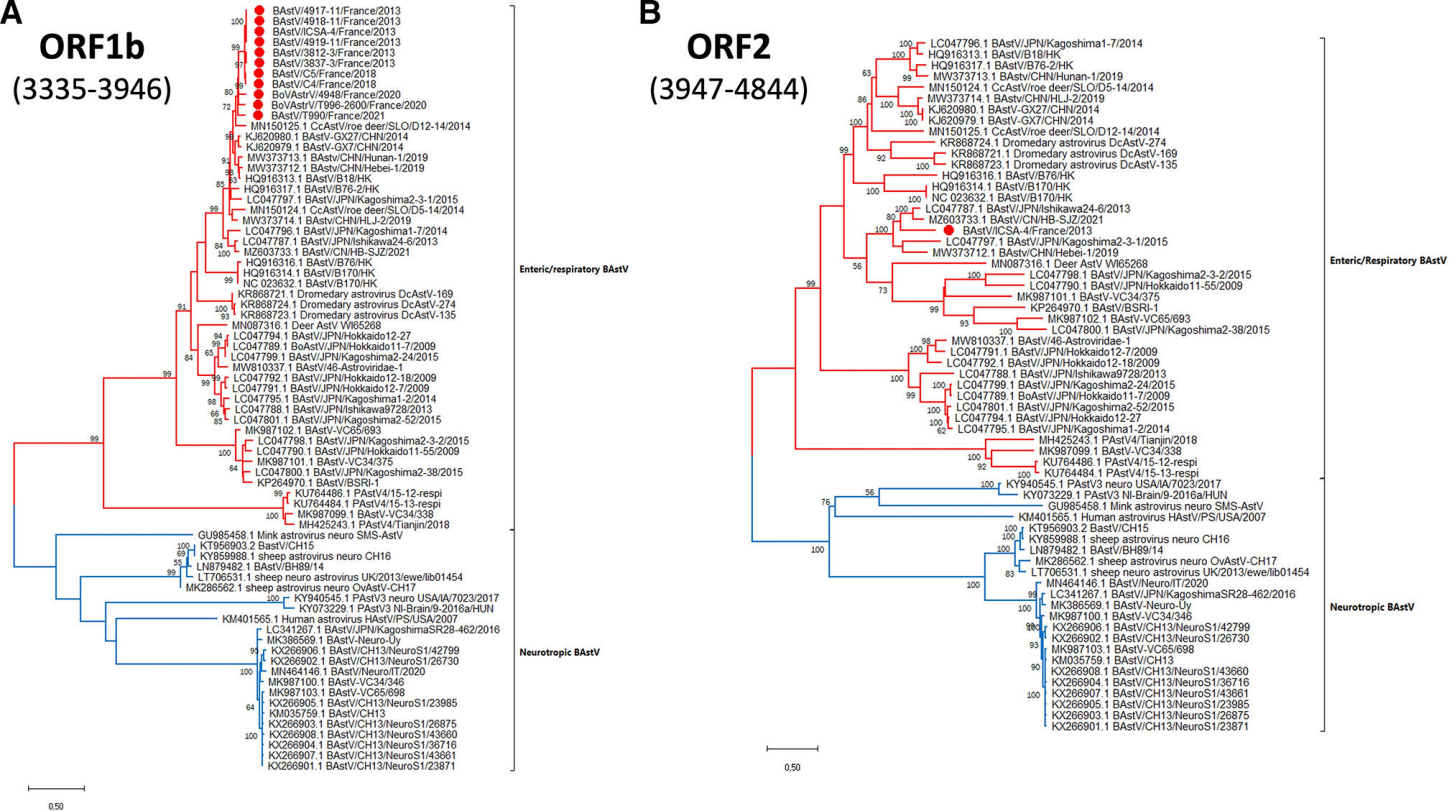
Maximum-Likelihood phylogenetic tree of the (A) Rdrp (within the ORF1b) and (B) a partition of the conserved region of the ORF2 of BAstV. The sequences generated in this work are highlighted with a red dot. The tree was constructed using 1,000 bootstrap replicates. The scale bar represents the number of nucleotide substitutions/site/year.

Before carrying out phylogenetic analyses, we searched for recombination events using the GARD algorithm. Sequence alignments were created based on publicly available BAstV whole genome sequences and on sequences obtained from the samples collected in France. We detected, in total, six recombination breakpoints throughout the BastV genome in our samples, with the best breakpoint locations at nucleotides: 1,884, 2,620, 3,334, 3,946, 4,844, and 5,881. However, they did not alter the global clustering of our full genome sequence, BAstV/ICSA-4/France/2013, always in the same sub-clade as BAstV/JPN/Ishikawa24-6/2013 and BAstV/CN/HB-SJZ/2021 (two Asian enteric BAstV), nor the branch topology of the trees. For phylogenetic reconstruction, we selected two different regions encompassing the breakpoint placements inferred by the GARD algorithm: the first on the Rdrp (nt 3,335–3,946), and the second one on the conserved region of the ORF2 (nt 3,947–4,844), as shown in Fig. 1. Both inferred phylogenetic trees displayed an equivalent branch topology. Our results indicate that two completely divergent clades for neurotropic and enteric BAstV exist, suggesting that certain molecular determinants are linked to tissue tropism. Our phylogenetic analysis indicates that BAstV detected in French calves cluster within the enteric BAstV group (Fig. 1). Moreover, BAstV detected in France are related to enteric strains already described in cattle in Asia yet displaying only between 85% and 95% nucleotide identity (Table 2). In addition, the French sequences belong to the same clade of BAstV that were identified in yaks, roe deer, and camelids, underlining the interspecies transmission potential of this virus. To confirm the absence of coinfecting neurotropic BAstV in our respiratory samples, we assembled BAL and NS NGS libraries using CH13 and CH15 as reference genomes. No reads were assembled when using the neurotropic sequences as reference genomes, indicating only the presence of enteric BAstV in cattle respiratory samples.

## DISCUSSION

BRD is a burden for cattle farms worldwide and viruses are known etiological agents that play an important role as triggers for secondary superinfections, often leading to pneumonia (37). The advent of NGS sequencing has allowed researchers to significantly improve pathogen detection in clinical specimens, both in human and veterinary medicine (38). In the literature, BAstV were previously detected in nasal swabs collected from calves displaying respiratory disease in one study in the United States (22). In our study, we detected BAstV by metagenomic sequencing in both the upper and lower respiratory tracts of animals displaying respiratory signs. In addition, we confirmed BAstV presence in cattle lungs in further clinical specimens by RT-qPCR. These data suggest that BAstV could potentially represent BRD agents that were never considered before; however, in our study, we did not include a healthy control group. The detection of BAstV in the lower respiratory tract might not be directly linked to respiratory signs in calves. BRD agents are mostly detected in coinfection in the field, making it challenging to differentiate the clinical signs induced by each single pathogen. Indeed, the majority of BAstV in lungs were detected in coinfection with other BRD agents, which are known to be pathogenic (mainly BCoV, but also BRSV). Therefore, the role of BAstV alone in BRD onset cannot be concluded. Coinfections between multiple pathogens are known to affect the morbidity and pathogen shedding during respiratory infections. In the literature, different examples of how the interaction between two respiratory pathogens impacts viral shedding and clinical signs have been described (37). Therefore, BastV detection in the lower respiratory tract could be due to the interaction with other coinfecting pathogens that might facilitate its shedding.

By NGS, we also detected BRAV and BRBV in NS and BAL of ill animals, and after *in vivo* challenge, BRBV was shown to cause upper respiratory tract infection in calves (39). BAstV could have a similar pathogenesis to BRBV but further samplings in healthy and sick cattle are warranted to better understand their involvement in triggering bovine pneumonia.

So far, no data about BAstV tropism in the respiratory tract are available in the literature. Different scenarios about BAstV detection in the lungs of calves with BRD could be hypothesized: BAstV could be a pathogen with a primary enteric tropism that reaches the respiratory tract in a second stage of the infection; however, the opposite situation cannot be ruled out. Currently, the lack of adequate models for virus isolation prevents us from further studying BAstV tropism *in vivo*.

*Mamastrovirus* was successfully isolated from different species, including humans, rabbits, and swine (40 – 43). *Avastrovirus* isolation has also been reported in different studies (44, 45). Attempts to isolate BAstV in our laboratory have failed so far, preventing us from further characterizing the clinical significance of BAstV in respiratory disease. This could be due to the cell lines used for virus isolation, the presence of co-infecting viruses in lungs and nostrils, or the degradation of clinical samples after years of storage. However, after the first cell passage, Cq values of 29 and 30 were observed for some samples. Future works should focus on the development of a suitable *in vitro* model to allow the generation of viral stocks and, therefore, the study of BAstV replication, tropism, and pathogenesis *in vivo*.

Our phylogenetic results agree with those previously published and suggest that different BAstV genotypes could be responsible for encephalitis and diarrhea. The dual enteric and neurotropic tropism observed in cattle was also described in other species, including minks (46, 47), swine (48, 49), sheep (50, 51), and humans (52 – 54). In cats and dogs, AstV infection has been linked only to gastroenteric disease so far (55 – 58). In the respiratory samples sequenced by NGS included in our study, we excluded the presence of neurotropic BAstV; however, the presence of other genotypes that were not detected by our RT-qPCR assay cannot be ruled out, as Astroviruses display high mutation and recombination rates. Additional studies are warranted to determine the relationship between genotype and clinical signs. More surveillance studies are necessary to better understand BAstV transmission dynamics and to better characterize circulating strains. Despite the detection of BAstV in the upper and lower respiratory tract of calves with BRD, their role as causal agents for respiratory disease remains to be elucidated.

## Data Availability

The BAstV sequences generated and analyzed in this study are available in the GenBank repository with accession numbers from OR261079 to OR261089.

## SUPPLEMENTAL MATERIAL

Figure S1 spectrum.03026-23-s0001.docx Locations of BAL and NS collected in two French regions.

Figure S2 spectrum.03026-23-s0002.docx Result of the ORF prediction analysis of the whole genome of BAstV/ICSA-4/France/2013.

ASM does not own the copyrights to Supplemental Material that may be linked to, or accessed through, an article. The authors have granted ASM a non-exclusive, world-wide license to publish the Supplemental Material files. Please contact the corresponding author directly for reuse.
