# Supplementary material for: Identification of Astrovirus in the virome of the upper and lower respiratory tracts of calves with acute signs of bronchopneumonia
Source: Microbiol Spectr. 2023 Nov 20;11(6):e03026-23. doi: 10.1128/spectrum.03026-23 (PMC10714732; doi:10.1128/spectrum.03026-23)
Supplement: Figure S1 — Locations of BAL and NS collected in two French regions. [file spectrum.03026-23-s0001.docx]

**Supplementary material**

**Figure S1.** Locations of BAL and NS collected in two French regions. The samples were collected during respiratory outbreaks in 2013-2016 and 2018 in calves’ farms that were 150 km radius from the city of Toulouse (depicted with a black triangle) in the Occitanie region, (South-West of France). In 2020-2021, the samples were collected in farms located 70 km radius from Carhaix-Plouguer (depicted with a black triangle) in Bretagne region (North-West of France).
