# Supplementary material for: Identification of Astrovirus in the virome of the upper and lower respiratory tracts of calves with acute signs of bronchopneumonia
Source: Microbiol Spectr. 2023 Nov 20;11(6):e03026-23. doi: 10.1128/spectrum.03026-23 (PMC10714732; doi:10.1128/spectrum.03026-23)
Supplement: Figure S2 — Result of the ORF prediction analysis of the whole genome of BAstV/ICSA-4/France/2013. [file spectrum.03026-23-s0002.docx]

**Supplementary material**

**Figure S2.** Result of the ORF prediction analysis of the whole genome of BAstV/ICSA-4/France/2013. ORFs were predicted using NCBI ORF finder (<https://www.ncbi.nlm.nih.gov/orffinder>).
